# Modeling of the Bioactivation of an Organic Nitrate by a Thiol to Form a Thionitrate Intermediate

**DOI:** 10.3390/molecules22010019

**Published:** 2016-12-25

**Authors:** Tsukasa Sano, Keiichi Shimada, Yohei Aoki, Takayuki Kawashima, Shohei Sase, Kei Goto

**Affiliations:** 1 Department of Chemistry, School of Science, Tokyo Institute of Technology, 2-12-1 Ookayama, Meguro-ku, Tokyo 152-8551, Japan; tsukasano8.24@gmail.com (T.S.); organic_12_05@yahoo.co.jp (Y.A.); ssase@chem.titech.ac.jp (S.S.); 2 Department of Chemistry, Graduate School of Science, The University of Tokyo, 7-3-1 Hongo, Bunkyo-ku, Tokyo 113-0033, Japan; shimak@cronos.ocn.ne.jp (K.S.); t.kawashima304@gmail.com (T.K.)

**Keywords:** thionitrates, reactive intermediates, kinetic stabilization, organic nitrates, nitric oxide, biotransformation, X-ray crystallographic analysis

## Abstract

Thionitrates (R–SNO_2_) have been proposed as key intermediates in the biotransformation of organic nitrates that have been used for the clinical treatment of angina pectoris for over 100 years. It has been proposed and widely accepted that a thiol would react with an organic nitrate to afford a thionitrate intermediate. However, there has been no example of an experimental demonstration of this elementary chemical process in organic systems. Herein, we report that aryl- and primary-alkyl-substituted thionitrates were successfully synthesized by the reaction of the corresponding lithium thiolates with organic nitrates by taking advantage of cavity-shaped substituents. The structure of a primary-alkyl-substituted thionitrate was unambiguously established by X-ray crystallographic analysis.

## 1. Introduction

**Scheme 1:**
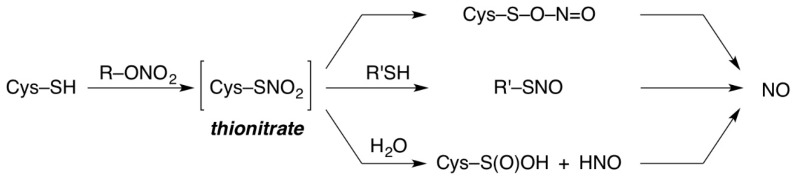
Proposed thiol-dependent pathways of biotransformation of organic nitrates.

**Scheme 2:**
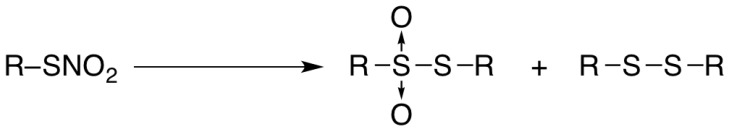
Bimolecular decomposition of thionitrates.

Organic nitrates such as nitroglycerin and isosorbide-5-mononitrate, have been widely used for the treatment of angina pectoris and congestive heart failure since the first clinical application of nitroglycerin in the late 19th century. They are regarded as nitric oxide (NO) prodrugs, and it has been assumed that they are converted to NO or an NO congener, which leads to vasorelaxation [1,2,3,4]. Although the mechanism of biotransformation of organic nitrates is still not fully understood, the potential chemical mechanism is a thiol-dependent pathway, for which several reaction pathways have been proposed as shown in Scheme 1 [5,6]. Importantly, all of them have the same first step in common, i.e., the formation of a thionitrate by the reaction of a cysteine thiol (Cys-SH) in protein with an organic nitrate. However, very little chemical evidence has been available for this elementary chemical transformation [7,8]. Recently, a thionitrate intermediate, which was formed in the active site of aldehyde dehydrogenase-2 by soaking with nitroglycerin, has been observed by protein X-ray crystallographic analysis and also detected by mass spectrometry [9]. In the artificial systems, thionitrates are highly reactive species and easily undergo bimolecular decomposition (Scheme 2) [10,11,12]. Hence, model studies on the chemical processes involving R–SNO_2_ have been difficult and scarcely investigated [12,13,14].

**Figure 1:**
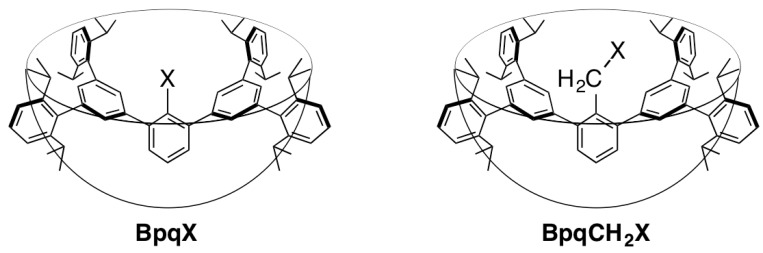
Molecular cavities utilized for the present model study.

In the course of our studies on biologically relevant highly reactive species containing sulfur and selenium, we have developed various cavity-shaped substituents and demonstrated that these substituents are very effective for kinetic stabilization of highly reactive species that otherwise undergo facile bimolecular decomposition [13,14,15,16,17,18,19,20,21,22]. Herein, we report a model study on the bioactivation of an organic nitrate by a thiol, which is the fundamental chemical process in the thiol-dependent biotransformation of nitrates. By taking advantage of the cavity-shaped aromatic and primary-alkyl substituents (Figure 1), denoted as Bpq and BpqCH_2_, respectively, we demonstrated that thionitrates are formed by the reaction of the corresponding thiolates with organic nitrates. The first crystallographic analysis of a primary-alkyl-substituted thionitrate is also presented.

## 2. Results and Discussion

**Scheme 3:**
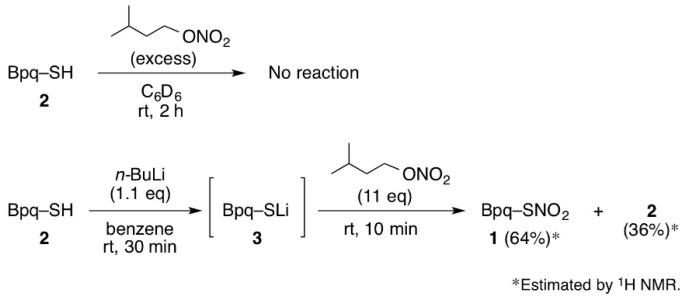
Reactions of thiol **2** with isoamyl nitrate.

Several stable thionitrates have been synthesized and isolated by taking advantage of kinetic stabilization utilizing appropriate bulky substituents such as tertiary alkyl groups [10,11,16] and cavity-shaped aromatic groups [17,23]. We previously reported the synthesis of a stable aryl thionitrate, BpqSNO_2_ (**1**), by the reaction of the corresponding *S-*nitrosothiol with an excess amount of *t-*BuONO or N_2_O_4_ [17]. Thus, we first employed the Bpq group for a model study on the bioactivation of organic nitrates. Treatment of thiol **2** bearing a Bpq group with an excess amount of isoamyl nitrate in C_6_D_6_ resulted in no reaction (Scheme 3). To increase the nucleophilicity of the sulfur, **2** was deprotonated with *n-*BuLi to generate lithium thiolate **3**, which was then allowed to react with an excess amount of isoamyl nitrate in benzene. This reaction sequence successfully afforded thionitrate **1** (64%) together with thiol **2** (36%). This is the first experimental demonstration of the formation of a thionitrate by the reaction of a thiolate with an organic nitrate.

**Scheme 4:**
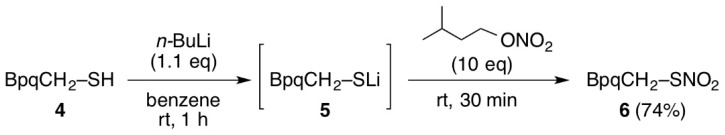
Synthesis of thionitrate **6** by the reaction of thiolate **5** with isoamyl nitrate.

**Scheme 5:**
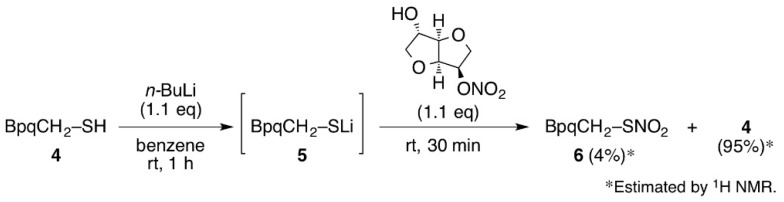
Formation of **6** by the reaction of thiolate **5** with ISMN.

As a model compound for naturally occurring cysteine-derived thionitrates (Cys-SNO_2_), a primary-alkyl-substituted thionitrate is considered to be the most relevant. However, the steric demands of usual primary-alkyl groups are too small to protect such reactive species, and there has been no example of the synthesis of a thionitrate bearing a primary-alkyl group. Meanwhile, we have recently developed an effective primary-alkyl steric protection group, a BpqCH_2_ group (Figure 1), with a cavity-shaped framework and succeeded in the isolation of reactive species such as a sulfenic acid [18], a sulfenyl iodide [19], a selenenic acid [20], and a selenenyl iodide [21] by utilizing this substituent. Thus, a model study by utilizing the BpqCH_2_ group was examined. When lithium thiolate **5**, prepared by the reaction of BpqCH_2_SH (**4**) with *n-*BuLi, was treated with an excess amount of isoamyl nitrate in benzene, the corresponding thionitrate **6** was formed almost quantitatively and isolated as stable colorless crystals in 74% yield after recrystallization (Scheme 4). Characterization of **6** was performed by NMR and IR spectroscopies and elemental analysis. In the IR spectrum of **6**, asymmetric and symmetric NO_2_ vibrational bands were observed at 1531 and 1297 cm^−1^, respectively, which are almost identical to those of tertiary-alkyl-substituted thionitrates [16]. The ^1^H-NMR spectrum of **6** exhibited a singlet due to the methylene protons in the CH_2_SNO_2_ moiety at 5.10 ppm, which is shifted downfield by more than 1 ppm relative to that of thiol **4** (3.91 ppm). In the ^13^C-NMR spectrum of **6**, the methylene carbon resonates at 41.3 ppm, which is also shifted downfield in comparison with **4** (30.5 ppm). The reaction of thiolate **5** with isosorbide-5-mononitrate (ISMN), which is widely used as a drug in treatment of angina pectoris, also afforded thionitrate **6** (Scheme 5), albeit in low yield (4%). Since thiol **4** was recovered in 95% yield in this reaction, it is probable that most of **5** was protonated and deactivated by the OH group in ISMN, resulting in the low yield of **6**. 

**Figure 2:**
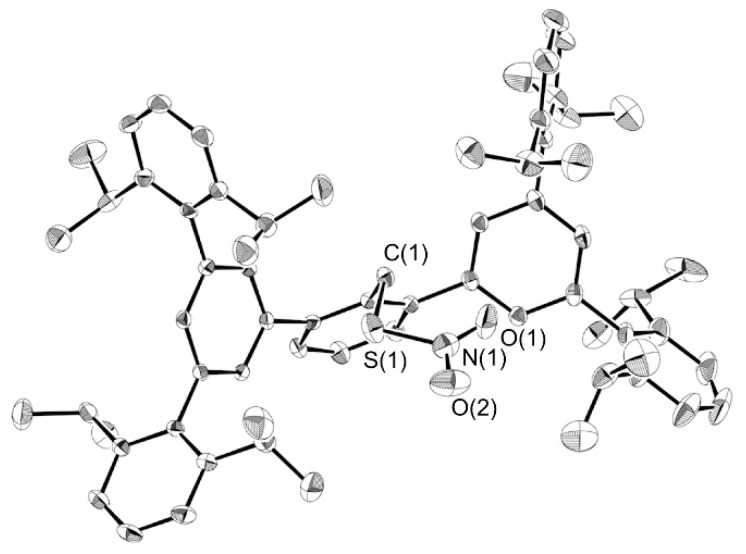
Oak Ridge thermal ellipsoid plot (ORTEP) drawing of **6** (50% probability).

The structure of **6** was unambiguously established by X-ray crystallographic analysis (Figure 2), presenting the first example of the crystallographic analysis of a primary-alkyl-substituted thionitrate. The CH_2_SNO_2_ moiety is incorporated within the cavity and effectively protected from the bimolecular decomposition by the peripheral moiety of the substituent. Selected structural parameters of **6** are summarized in Table 1, together with those of the aryl-substituted thionitrates **1** [14] and the tertiary-alkyl-substituted thionitrate **7** [16] reported by us as well as those of the aryl derivative **8** [24] reported by Itoh et al. In the crystalline state, **6** features disorder of the SNO_2_ moiety with respect to a mirror plane perpendicular to the central benzene ring in the ratio of 0.8:0.2, both of which possess a similar structure. The structural parameters of the major component are discussed in the following. The CH_2_SNO_2_ moiety is well incorporated within the cavity of the Bpq group. The C–S–N–O dihedral angles are 4.8(5)° and –176.0(4)°, showing that the C–SNO_2_ moiety is almost coplanar. The C–S bond length (1.803(3) Å) in **6** is slightly longer than those in the aromatic derivatives **1** (1.7651(15) Å) and **8** (1.764(2) Å) and similar to that of the tertiary-alkyl derivative **7** (1.789(6) Å). The C–S–N bond angles of **6** (100.5(2)°) are similar to those of **1** (100.47(7)°) and **8** (99.75(10)°) yet much smaller than that of **7** (107.9(5)°). The enlargement of the C–S–N bond angle was observed only in **7**, which is probably due to the steric repulsion between the SNO_2_ moiety and *m*-terphenyl groups. The structure of the primary-alkyl-substituted compound **6** shown here is expected to serve as reference data for X-ray crystallographic analysis of protein–SNO_2_.

## 3. Materials and Methods

Unless otherwise stated, all operations were performed in a MBraun UNIlab glovebox an argon atmosphere, in a Miwa 1ADB-3KTG glovebox under a nitrogen atmosphere, or by using high-vacuum and standard Schlenk techniques under an argon atmosphere. Benzene (anhydrous) was purchased from Kanto Chemical (Tokyo, Japan) and distilled from benzophenone ketyl prior to use. Other chemicals were purchased from commercial sources and used as received. ^1^H-NMR spectra were recorded on a JEOL ECX-500, a JEOL ECX-400, or a JEOL ECS-400 spectrometer (JEOL, Tokyo, Japan), and the chemical shifts of ^1^H are referenced to the residual proton signal of CDCl_3_ (δ 7.25). ^13^C-NMR spectra were recorded on a JEOL ECX-500 or a JEOL ECX-400 spectrometer (JEOL, Tokyo, Japan), and the chemical shifts of ^13^C are referenced to the signal of CDCl_3_ (δ 77.0). All spectra were assigned with the aid of DEPT (distorsionless enhancement by polarization transfer), COSY (correlated spectroscopy), HMQC (heteronuclear multi quantum correlation), and HMBC (heteronuclear multiple bond correlation) NMR experiments. IR spectra were recorded on a JASCO FT/IR-4100 (JASCO, Tokyo, Japan) by utilizing a JASCO ATR Pro550S unit. Melting points were measured with a Yanaco MP-S3 (Yanaco, Tokyo, Japan)and are uncorrected.

### 3.1. The Reaction of Thiol **2** with Isoamyl Nitrate

To a solution of thiol **2** (3.5 mg, 3.9 µmol) in C_6_D_6_ (0.6 mL) was added isoamyl nitrate (30 µL, 0.23 mmol) at ambient temperature. The reaction was monitored by ^1^H-NMR spectroscopy in a screw-capped NMR tube, and no reaction was observed for 2 h. 

### 3.2. Synthesis of Thionitrate **1**

*n-*BuLi (1.6 mol/L in hexane, 0.30 mL, 0.48 mmol) was diluted with benzene (3 mL). To a solution of thiol **2** (18.3 mg, 20 µmol) in benzene (6 mL) was added the diluted *n-*BuLi solution (0.15 mL, 22 µmol). The reaction mixture was stirred for 30 min at ambient temperature, and then isoamyl nitrate (30 µL, 0.23 mmol) was added at ambient temperature. After 10 min, the solvent was removed in vacuo, and the residue was washed with hexane to afford the mixture of thionitrate **1** [17] and thiol **2**. The yields of **1** and **2** were estimated to be 64% and 36%, respectively, by ^1^H-NMR spectroscopy.

### 3.3. Synthesis of Thionitrate **6**

In a glovebox under an argon atmosphere, to a solution of thiol **4** (20.3 mg, 22.1 µmol) [18] in benzene (6.6 mL) in a Schlenk flask with a J-young valve was added *n-*BuLi (56 mM in hexane, 434 µL, 24 µmol) at ambient temperature. After 1 h, the flask was carefully sealed and transferred to a glovebox under a nitrogen atmosphere. To the reaction mixture was added isoamyl nitrate (27 µL, 0.20 mmol) at ambient temperature. After 30 min, the resulting yellow solution was evaporated in vacuo, followed by recrystallization from hexane to afford **6** (14.2 mg, 16 µmol, 74%) as colorless crystals. 

**6**: colorless crystals; mp 203.5–204.4 °C (dec); ^1^H-NMR (400 MHz, CDCl_3_) δ 1.07 (d, *J* = 6.9 Hz, 24H), 1.13 (d, *J* = 6.9 Hz, 24H), 2.80 (sept, *J* = 6.9 Hz, 8H), 5.10 (s, 2H), 7.06 (t, *J* = 1.5 Hz, 2H), 7.14–7.22 (m, 12H), 7.27-7.50 (m, 7H); ^13^C-NMR (126 MHz, CDCl_3_) δ 24.0 (q), 24.1 (q), 30.6 (d), 41.3 (t), 122.5 (d), 128.0 (d), 128.4 (d), 128.6 (d), 129.8 (d), 130.5 (d), 138.5 (s), 139.8 (s), 141.1 (s), 143.9 (s), 146.67 (s), 146.72 (s); IR (ATR) *υ* 1531 cm^−1^ (asym-NO_2_), 1297 cm^−1^ (sym-NO_2_). Anal. Calcd for C_67_H_79_NOS: C, 83.61; H, 8.27, N, 1.46; S, 3.33. Found: C, 83.37; H, 8.38; N, 1.33; S, 3.20.

### 3.4. Formation of **6** by the Reaction with Isosorbide-5-Mononitrate (ISMN)

In a glovebox under an argon atmosphere, to a solution of thiol **4** (18.5 mg, 20.2 µmol) in benzene (6 mL) in a Schlenk flask with a J-young valve was added *n-*BuLi (56 mM in hexane, 436 µL, 24 µmol) at ambient temperature. After 1 h, the flask was carefully sealed and transferred to a glovebox under a nitrogen atmosphere. To the reaction mixture was added a solution of ISMN (4.3 mg, 22 µmol) in benzene (1 mL) at ambient temperature. After 30 min, the reaction mixture was evaporated in vacuo. The ^1^H-NMR analysis of the crude mixture confirmed the formation of **6** (4%) together with **4** (95%).

### 3.5. X-Ray Crystallography

Single crystals of **6**·1.5CHCl_3_ were grown in their hexane-CHCl_3_ solution. A colorless crystal of **6**·1.5CHCl_3_ was mounted on a loop. All measurements were made on a Rigaku/MSC Mercury CCD (Charge Coupled Device) diffractometer (Rigaku, Tokyo, Japan) with graphite-monochromated Mo-Kα radiation (λ = 0.71070 Å) at –153 °C. The structures were solved by the direct method and refined by full-matrix least squares on *F^2^*using SHELXL 97 [25]. Crystal Data for **6**·1.5CHCl_3_ (*M* = 1141.42 g/mol): triclinic, space group *P*-1, *a* = 11.6005(15) Å, *b* = 15.8742(14) Å, *c* = 18.923(2) Å, *α* = 99.399(3)°, β = 105.607(4)°, γ = 100.004(3)°, *V* = 3223.0(6) Å^3^, *Z* = 2, *D*_calc_ = 1.176 g/cm^3^, 20857 reflections measured, 11040 unique (*R*_int_ = 0.0148). The final *R*_1_ was 0.0941 (*I* > 2*σ*(*I*)) and *wR*_2_ was 0.2475 (all data). CCDC 1518311 contains the supplementary crystallographic data for this paper. These data can be obtained free of charge via http://www.ccdc.cam.ac.uk/conts/retrieving.html (or from the CCDC, 12 Union Road, Cambridge CB2 1EZ, UK; Fax: +44 1223 336033; E-mail: deposit@ccdc.cam.ac.uk)

## 4. Conclusions

Formation of a thionitrate by the reaction of a thiolate with an organic nitrate was experimentally demonstrated for the first time by taking advantage of molecular cavities. The X-ray crystallographic analysis of a primary-alkyl-substituted thionitrate was also established. The present results provide chemical corroboration to the formation of a thionitrate intermediate proposed for the first step in the thiol-mediated bioactivation of an organic nitrate, which has long been suggested but not evidenced. Further investigations on the modeling of the bioactivation processes of organic nitrates are currently in progress.

## Figures and Tables

**Table 1:**
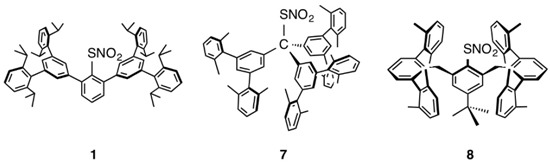
Selected bond lengths and angles for thionitrates.

	**6 (this work)**	**1** [14]	**7** [16]	**8** [24]
Bond lengths (Å)				
S(1)–N(1)	1.780 (6)	1.7898 (17)	1.746 (9)	1.795 (2)
N(1)–O(1)	1.205 (8)	1.2180 (19)	1.229 (9)	1.215 (3)
N(1)–O(2)	1.211 (6)	1.213 (2)	1.239 (9)	1.228 (3)
C(1)–S(1)	1.803 (3)	1.7651 (15)	1.789 (6)	1.764 (2)
Bond angles (°)				
S(1)–N(1)–O(1)	121.7 (4)	120.25 (12)	114.1 (9)	121.19 (17)
S(1)–N(1)–O(2)	114.1 (9)	113.28 (13)	119.7 (9)	113.48 (18)
O(1)–N(1)–O(2)	124.00 (57)	126.44 (17)	126.2 (11)	125.3 (2)
C(1)–S(1)–N(1)	100.5 (2)	100.47 (7)	107.9 (5)	99.75 (10)

## Supplementary Materials

The following are available online at http://www.mdpi.com/1420-3049/22/1/19/s1.
