# Supplementary material for: Modeling of the Bioactivation of an Organic Nitrate by a Thiol to Form a Thionitrate Intermediate
Source: Molecules. 2016 Dec 25;22(1):19. doi: 10.3390/molecules22010019 (PMC6155724; doi:10.3390/molecules22010019)
Supplement: Supplementary file 1 [file molecules-22-00019-s001.pdf]

Supplementary Materials: Modeling of the Bioactivation of an Organic Nitrate by a Thiol to Form a Thionitrate Intermediate

Tsukasa Sano, Keiichi Shimada, Yohei Aoki, Takayuki Kawashima, Shohei Sase and Kei Goto

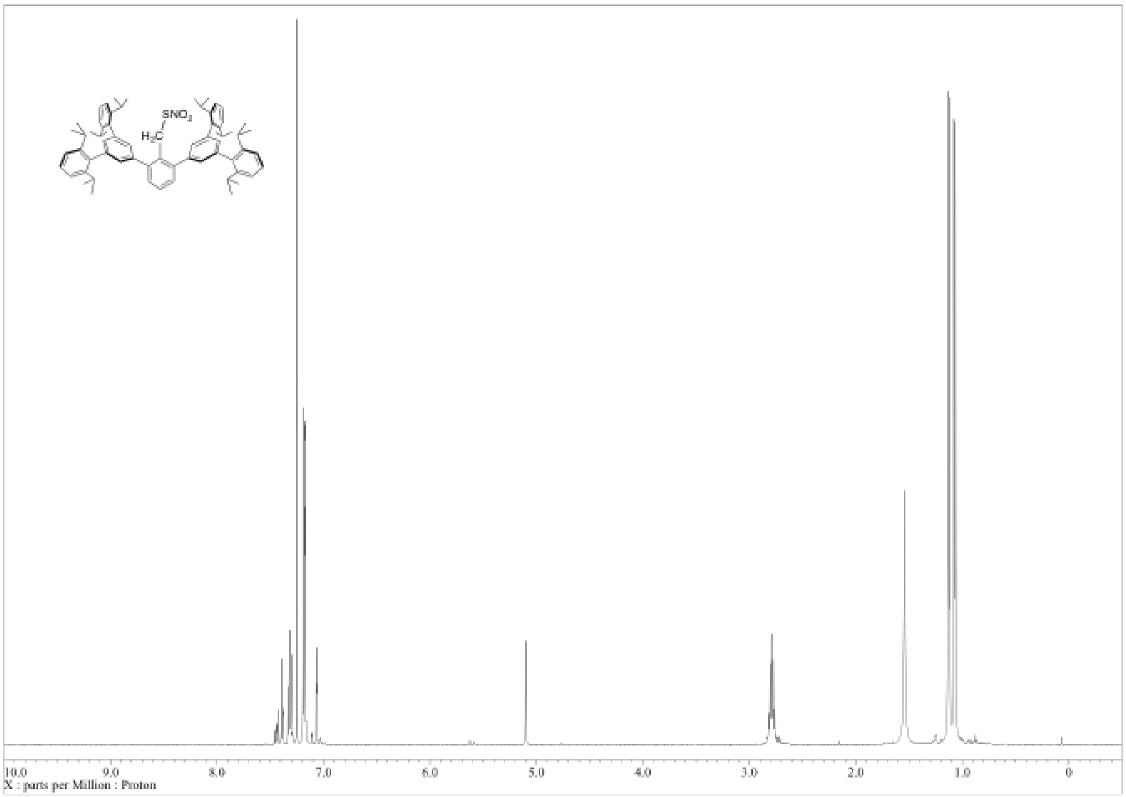

Figure S1. ^{1}H NMR (500 MHz) spectrum of compound **6** in $CDCl_3$.

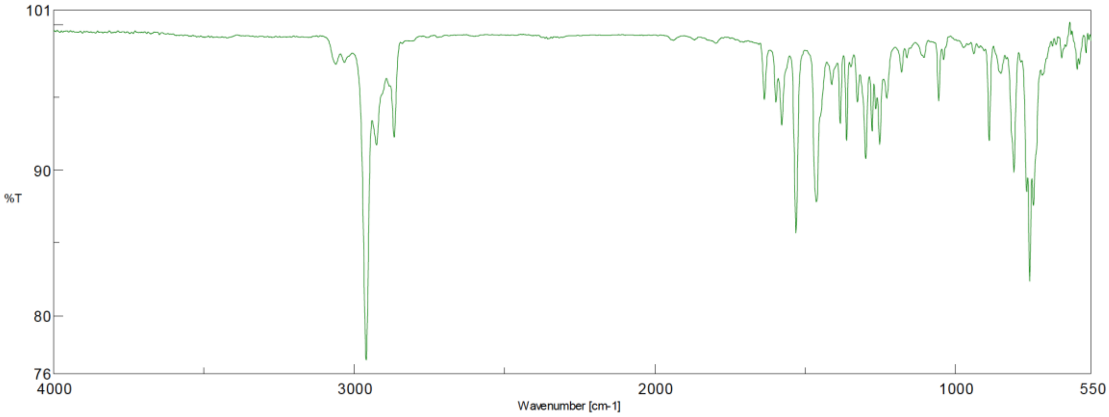

Figure S2. IR (ATR) spectrum of compound **6**.
